# Lifestyle factors associated with prevalent and exacerbated musculoskeletal pain after the Great East Japan Earthquake: a cross-sectional study from the Fukushima Health Management Survey

**DOI:** 10.1186/s12889-020-08764-9

**Published:** 2020-05-13

**Authors:** Hiroshige Jinnouchi, Tetsuya Ohira, Hironobu Kakihana, Ko Matsudaira, Masaharu Maeda, Hirooki Yabe, Yuriko Suzuki, Mayumi Harigane, Hiroyasu Iso, Tomoyuki Kawada, Seiji Yasumura, Kenji Kamiya, on behalf of the Mental Health Group of the Fukushima Health Management Survey

**Affiliations:** 1 Department of Hygiene and Public Health, Nippon Medical School, 1-1-5 Sendagi, Bunkyo-ku, Tokyo, 113-8602 Japan; 2 Public Health, Department of Social Medicine, Osaka University Graduate School of Medicine, 2-2 Yamadaoka, Suita, Osaka, 565-0871 Japan; 3 Department of Public Health Medicine, Faculty of Medicine, and Health Services Research and Development Center, University of Tsukuba, Tsukuba, 305-8575 Japan; 4 Department of Epidemiology, Fukushima Medical University School of Medicine, 1 Hikariga-oka, Fukushima, 960-1295 Japan; 5 Radiation Medical Science Center for the Fukushima Health Management Survey, Fukushima Medical University, 1 Hikariga-oka, Fukushima, 960-1295 Japan; 6 Department of Hygiene and Public Health, Osaka Medical College, 2-7 Daigakumachi, Takatsuki, Osaka, 569-8686 Japan; 7 Department of Medical Research and Management for Musculoskeletal Pain, 22nd Century Medical and Research Center, Faculty of Medicine, the University of Tokyo, 7-3-1 Hongo, Bunkyo-ku, Tokyo, 113-8655 Japan; 8 Department of Disaster Psychiatry, Fukushima Medical University School of Medicine, 1 Hikariga-oka, Fukushima, 960-1295 Japan; 9 Department of Neuropsychiatry, Fukushima Medical University School of Medicine, 1 Hikariga-oka, Fukushima, 960-1295 Japan; 10 Department of Adult Mental Health, National Center of Neurology and Psychiatry, National Institute of Mental Health, 4-1-1 Ogawa-Higashi, Kodaira, Tokyo, 187-8553 Japan; 11 Department of Public Health, Fukushima Medical University School of Medicine, 1 Hikariga-oka, Fukushima, 960-1295 Japan; 12 Research Institute for Radiation Biology and Medicine, Hiroshima University, 1-2-3 Kasumi, Minami-ku, Hiroshima City, Hiroshima, 734-8553 Japan

**Keywords:** Musculoskeletal pain, Lifestyle factor, The Great East Japan Earthquake

## Abstract

**Background:**

While the prevalence of post-disaster musculoskeletal pain has been documented, its associated disaster-related factors have not been investigated. This study was to investigate the association of lifestyle factors associated with musculoskeletal pain after the Great East Japan Earthquake.

**Methods:**

We conducted a cross-sectional study of 34,919 participants, aged 40–89 years, without any major disabilities at about 1 year after the disaster. The participants were asked about their musculoskeletal pain (low back and limb pain) and lifestyle factors: use of evacuation shelters or temporary housing at any point of time, job loss after the disaster, decreased income after the disaster, current smoking status, current drinking status, lack of sleep, regular exercise, and participation in recreational or community activities. Furthermore, psychological factors, such as traumatic reactions, psychological distress, and uncomfortable symptoms, affecting musculoskeletal pain were assessed. We used multinomial logistic regression analysis to calculate odds ratios of each lifestyle factor for prevalent and prevalent plus exacerbated musculoskeletal pain.

**Results:**

Musculoskeletal pain prevalence was 32.8%: 27.6% for prevalent and 5.2% for prevalent plus exacerbated musculoskeletal pain. Multivariable adjusted odds ratios and 95% confidence intervals of lifestyle factors associated with prevalent and prevalent plus exacerbated musculoskeletal pain were as follows: shelter use (prevalent: 1.02, 0.96–1.08; exacerbated: 1.44, 1.29–1.60), job loss (prevalent: 1.03, 0.96–1.10; exacerbated: 1.30, 1.16–1.47), decreased income (prevalent: 1.13, 1.05–1.21; exacerbated: 1.29, 1.14–1.45), current heavy drinking (prevalent: 1.33, 1.21–1.47; exacerbated: 1.38, 1.14–1.68), insomnia (prevalent: 1.22, 1.15–1.29; exacerbated: 1.50, 1.36–1.65), exercising almost daily (prevalent: 0.83, 0.77–0.91; exacerbated: 0.80, 0.68–0.95), and participating in community activities often (prevalent: 0.83, 0.75–0.92; exacerbated: 0.76, 0.61–0.95).

**Conclusions:**

Prevalent and exacerbated musculoskeletal pain were inversely associated with exercising almost daily and participating in recreational or community activities sometimes or often, and positively associated with decreased income, current heavy drinking, and insomnia. Besides, the use of evacuation shelters or temporary housing/job loss was positively associated only with exacerbated musculoskeletal pain. These results suggest that post-disaster lifestyle factors are potentially associated with musculoskeletal pain. To achieve better post-disaster pain management, further studies are needed to confirm the consistency of these results in other disasters and to highlight the underlying causative mechanisms.

## Background

Disasters cause dramatic impacts on our life; the long-lasting impacts gradually affect people’s health. Musculoskeletal pain affects daily activities and quality of life [1–3]. For example, according to the Global Burden of Disease Survey in 2016, back pain is the leading cause of years lived with disability, followed by neck pain (6th) and osteoarthritis (12th) [3]. Additionally, musculoskeletal pain increases social economic costs due to such as increased absenteeism and presenteeism, early retirement, and medical service use [4]. However, extensive epidemiology of post-disaster musculoskeletal pain has not been established, and insufficient studies are investigating its associations with lifestyle.

Most previous post-disaster studies have mainly focused on the prevalence of pain. A retrospective study of 958 evacuees conducted about 1 month after the 2009 earthquake in L’Aquila, Italy, showed that 35% of the evacuees had some pain, and, unlike acute pain, chronic pain increased over the 0.5 to 1 month following the disaster [5]. A cross-sectional study of 71 evacuees from the Great East Japan Earthquake conducted about 18 months (1.5 years) after the disaster showed that 62% of evacuees experienced chronic pain in some body part, the most frequent ones being the lower back and limbs [6]. From these findings, it is evident that more than half the evacuees experience musculoskeletal pain after a disaster. Other previous studies, while not conducted in post-disaster situations, have reported that musculoskeletal pain and its prevalence, persistence, and/or exacerbation are associated with lifestyle factors such as low physical activity [7–9], drinking status [10], and insomnia [11, 12]. Depressive symptoms are also associated with musculoskeletal pain [13] and can influence persistent or exacerbated pain through central pain mechanisms particularly involving amygdala and hippocampus [14].

The Great East Japan Earthquake of 2011 forced approximately 160,000 people to leave their hometowns. The evacuees experienced not only the earthquake itself but also a tsunami and/or the indirect effects of a nuclear power plant accident. These disasters and experiences affected their living environment, relatives and friends, and socioeconomic status [15, 16]. There were also some changes seen between the pre- and post-disaster phases in lifestyle status among evacuees; for example, smoking, drinking, and physical activities [17] as well as psychological distress [18]. However, the relationships between post-disaster lifestyle and musculoskeletal pain, as well as proportions of prevalent, persistent, and/or exacerbated pain in mid-to-long term after the disaster, are not well known. It is, therefore, important that we identify the relationships between lifestyle factors and musculoskeletal pain to achieve better mid-to-long term health management in the future disaster.

The purpose of this study was to investigate the association of lifestyle factors with the prevalence and exacerbation of musculoskeletal pain after the Great East Japan Earthquake. We hypothesised that lifestyle factors (such as the use of a shelter) might be related to musculoskeletal pain after a disaster. We also hypothesised that disaster-related psychological stress may increase musculoskeletal pain.

## Methods

### Subjects

This cross-sectional study included 55,727 evacuees, aged 40–89 years (mean age, 61 years), who experienced the Great East Japan Earthquake, and who had taken the Mental Health and Lifestyle Survey as a part of the Fukushima Health Management Survey. Detailed information about the survey is described elsewhere [19, 20]. Briefly, the target population were all the residents who lived in the evacuation zones, which is a government-designated area (20 km radius) around the nuclear power plant. The earthquake occurred on 11 March 2011, and the study period was from 18 January 2012 to 31 October 2012. We sent a self-administered questionnaire on various lifestyle and mental health factors to all the residents. The overall response rate of the Mental Health and Lifestyle Survey was 40.7% [16].

**Fig. 1:**
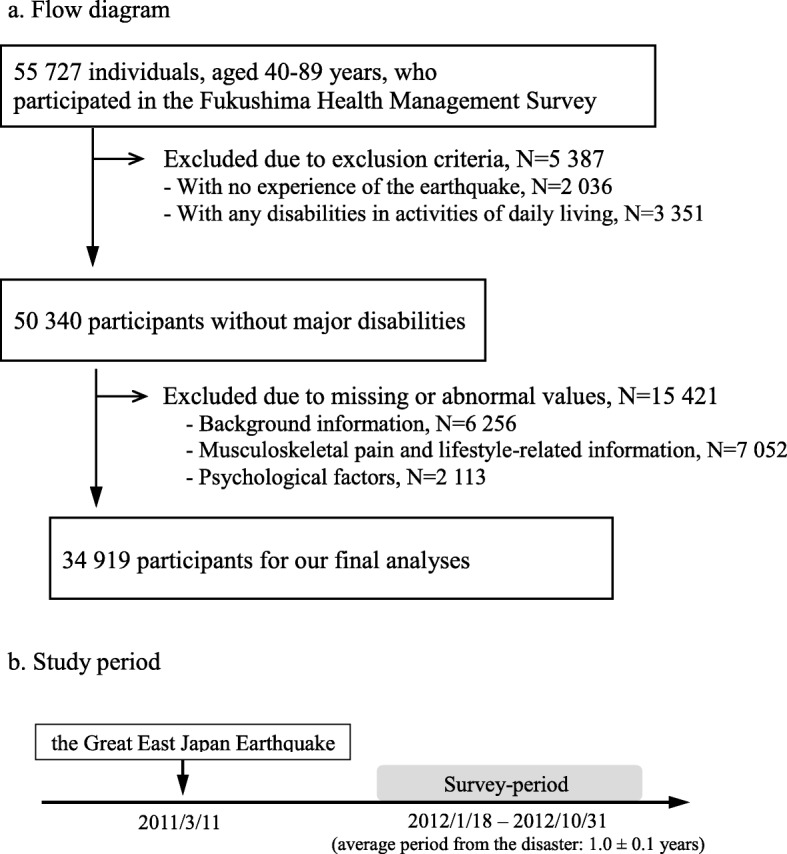
Diagram of participant enrollment, and selection

We defined an evacuee as a resident in evacuation areas who reported to have experienced the earthquake, including those who had experienced the tsunami and/or the indirect effects of the nuclear power plant accident. The exclusion criteria were as follows: not having experienced the earthquake (*n* = 2036); having any major disabilities in activities of daily living such as eating, dressing, going to the toilet, or shopping (*n* = 3351). Regarding the latter criteria, individuals with a disability are more likely to have musculoskeletal pain than those without a disability. Also, disability itself may impose physical limitations, social activity restrictions, and environmental needs. In this case, the effects of disability could not be independent of the other factors, and the associations of musculoskeletal pain with lifestyle factors may lead to be over- and under-estimations. Thus, we decided to exclude individuals having any major disabilities in the present analysis. We also excluded individuals with missing information or abnormal values for basic characteristics and medical history (*n* = 6256), musculoskeletal pain and lifestyle-related information (*n* = 7052), and psychological factors (*n* = 2113). Finally, we used 34,919 people (female, *n* = 18,156; male, *n* = 16,763) without major disabilities at about 1.0 ± 0.1 years (average ± standard deviation) after the disaster (Fig. 1). The Ethics Committee of Fukushima Medical University approved this survey protocol. We mailed a questionnaire stating its purpose and specified that it would be used for analysis. The participants subsequently provided their written consent to participate by returning the questionnaire.

### Musculoskeletal pain

We asked all participants to answer the multiple-choice question “In the past few days, have you experienced uncomfortable symptoms due to any diseases or injuries?”. If the participant responded “Yes” to low back pain or limb pain, we regarded that the participant had major musculoskeletal symptoms. The most common areas of musculoskeletal pains can be assumed the low back, knee, hip, ankle, foot, shoulder, elbow, wrist, and fingers (the latter eight are in the category of limb pain; our questionnaire did not include the neck) [21]. We further asked whether the pain was exacerbated after the disaster. The subjects were categorised into 3 groups: absent musculoskeletal pain (absent), prevalent musculoskeletal pain (prevalent), and prevalent plus exacerbated musculoskeletal pain (exacerbated).

### Lifestyle factors

We asked participants about lifestyle factors including the use of evacuation shelters or temporary housing at any point in time (shelter use: no/yes), having lost their job after the disaster (job loss: no/yes), decrease in income after the disaster (decreased income: no/yes), current smoking status (smoking status: never/past/current light smoking/current heavy smoking), current drinking status (drinking status: never/past/current light drinking/current heavy drinking), subjective lack of sleep (insomnia: no/yes), current regular exercise (regular exercise: almost none/1 time per week/2–4 times per week/almost daily), and participation in recreational or community activities (community activities: never or rarely/sometimes/often).

### Psychological factors

We assessed psychological factors that may affect musculoskeletal pain such as traumatic reactions (post-traumatic stress disorder check list: 44 points or more) [22, 23], psychological distress (Kessler psychological distress scale: 13 points or more) [24, 25], and uncomfortable symptoms (none/one/two or more symptoms except for musculoskeletal pain). Their uncomfortable symptoms were categorised into 4 groups as follows based on a study by Zijlema et al. (2013): cardiopulmonary/autonomic (palpitations, breathing trouble), gastrointestinal (diarrhoea/constipation, poor appetite), general (headaches, dizziness), and others (difficulty urinating) [26].

### Other confounding factors

We also gathered information regarding potential confounding factors such as age (40–64 years/65–74 years/75–89 years); sex (male/female); history of hypertension, diabetes, and dyslipidaemia (no/yes); education status (junior high school or less/high school/colleges/university or higher); having experienced the tsunami and/or indirect effects of the nuclear power plant accident (no/yes); house damage (none/partly destroyed/half or more destroyed); and loss of relatives or friends (no/yes).

### Statistical analysis

A generalised linear model was used to compare basic characteristics, psychological factors, and lifestyle factors between the groups (absent versus prevalent, absent versus exacerbated, and prevalent versus exacerbated). Because even trivial differences may be statistically significant due to our large sample, we regarded differences of ±3% or more between the groups in addition to *p*-values less than 0.05 as statistically significant to highlight the significance of our findings. Multinomial logistic regression analysis was used to calculate odds ratios and 95% confidence intervals of each lifestyle factor related to prevalent musculoskeletal pain and exacerbated musculoskeletal pain. Two multivariable adjusted models were constructed. Model 1 was the adjustment for background information (age, sex, history of hypertension/diabetes/dyslipidemia, and educational status) and disaster experience (tsunami/indirect nuclear power plant accident, house damage, loss of close person) and each lifestyle factor. Model 2 was the further adjustment for psychological factors (traumatic reactions and psychological distress) and the number of uncomfortable symptoms (none, one, and two or more) to the Model 1.

## Results

### Prevalence of musculoskeletal pain

The proportions of participants with prevalent and exacerbated musculoskeletal pain were 27.6 and 5.2%, respectively (Table 1). The proportion of participants with both low back pain and limb pain was greater in participants with exacerbated musculoskeletal pain than in those with prevalent musculoskeletal pain. According to additional stratified analyses, the prevalence of low back pain was 25.8% and that of limb pain was 17.2%. We also found that a greater prevalence was observed in women than in men and that prevalence increased with age, except for exacerbated low back pain (see the Supplemental Table 1).

**Table 1:** Prevalence of musculoskeletal pain and basic background information of 34,919 participants

	Musculoskeletal pain		
	Absent	Prevalent	Exacerbated
N	23,456	9650	1813
Age			
40 to 64 years	15,210 (64.8)	5713 (59.2)^a^	1077 (59.4)^ab^
65 to 74 years	5165 (22.0)	2266 (23.5)	440 (24.3)
75 to 89 years	3081 (13.1)	1671 (17.3)^a^	296 (16.3)^a^
Sex			
Male	11,690 (49.8)	4375 (45.3)^a^	698 (38.5)^ab^
Female	11,766 (50.2)	5275 (54.7)^a^	1115 (61.5)^ab^
History of disease			
Hypertension	10,358 (44.2)	5146 (53.3)^a^	988 (54.5)^a^
Diabetes	4551 (19.4)	2325 (24.1)^a^	453 (25.0)^a^
Dyslipidemia	8734 (37.2)	4486 (46.5)^a^	890 (49.1)^a^
Educational status			
Junior high school or less	5823 (24.8)	2853 (29.6)^a^	455 (25.1)^b^
High school	12,079 (51.5)	4773 (49.5)	917 (50.6)
Colleges/university or higher	5554 (23.7)	2024 (21.0)	441 (24.3)^b^
Type of disaster experience			
Tsunami	4603 (19.6)	2294 (23.8)^a^	473 (26.1)^a^
Indirect nuclear power plant accident	11,754 (50.1)	5743 (59.5)^a^	1198 (66.1)^ab^
House damage			
None	6296 (26.8)	2078 (21.5)^a^	311 (17.2)^ab^
Partly destroyed	13,811 (58.9)	5852 (60.6)	1109 (61.2)
Half or more destroyed	3349 (14.3)	1720 (17.8)^a^	393 (21.7)^a^
Loss of close person			
No	19,298 (82.3)	7362 (76.3)^a^	1286 (70.9)^ab^
Yes	4158 (17.7)	2288 (23.7)^a^	527 (29.1)^ab^
Pain region			
Low back pain only	–	4779 (49.5)	685 (37.8)^b^
Limb pain only	–	2124 (22.0)	319 (17.6)^b^
Both	–	2747 (28.5)	809 (44.6)^b^

Percentages are shown in parentheses. Statistically significant and observed ±3% or more difference compared to the Absent group (^a^), and to the Prevalent group (^b^); The total percentage does not always add up to 100

### Frequency analyses of lifestyle factors

Compared to absent pain, both participants with prevalent and exacerbated musculoskeletal pain showed significantly greater proportions of shelter use, job loss, insomnia, exercising almost not, and participating in community activities never or rarely. In comparison, a significantly smaller proportion were observed in exercising almost daily and participating in community activities sometimes or often. Decreased income, never smoking, never drinking, and current light drinking showed significantly differences only for exacerbated musculoskeletal pain (not for prevalent pain). (Table 2).

**Table 2:** Associated post-disaster lifestyle factors with prevalent and exacerbated musculoskeletal pain

	Musculoskeletal pain		
	Absent	Prevalent	Exacerbated
Shelter use			
No	15,931 (67.9)	6012 (62.3)^a^	923 (50.9)^ab^
Yes	7525 (32.1)	3638 (37.7)^a^	890 (49.1)^ab^
Job loss			
No	19,057 (81.2)	7438 (77.1)^a^	1257 (69.3)^ab^
Yes	4399 (18.8)	2212 (22.9)^a^	556 (30.7)^ab^
Decreased income			
No	19,106 (81.5)	7600 (78.8)	1374 (75.8)^ab^
Yes	4350 (18.5)	2050 (21.2)	439 (24.2)^ab^
Smoking status			
Never	12,862 (54.8)	5380 (55.8)	1086 (59.9)^ab^
Past	5848 (24.9)	2438 (25.3)	408 (22.5)
Current light smoking	2374 (10.1)	901 (9.3)	147 (8.1)
Current heavy smoking	2372 (10.1)	931 (9.6)	172 (9.5)
Drinking status			
Never	11,075 (47.2)	4664 (48.3)	916 (50.5)^a^
Past	727 (3.1)	367 (3.8)	71 (3.9)
Current light drinking	8976 (38.3)	3429 (35.5)	622 (34.3)^a^
Current heavy drinking	2678 (11.4)	1190 (12.3)	204 (11.3)
Insomnia			
No	15,728 (67.1)	4677 (48.5)^a^	645 (35.6)^ab^
Yes	7728 (32.9)	4973 (51.5)^a^	1168 (64.4)^ab^
Regular exercise			
Almost not	11,071 (47.2)	4783 (49.6)^a^	915 (50.5)^a^
1 time per week	3373 (14.4)	1387 (14.4)	240 (13.2)
2–4 times per week	4994 (21.3)	2117 (21.9)	422 (23.3)
Almost daily	4018 (17.1)	1363 (14.1)^a^	236 (13.0)^a^
Community activities			
Never or rarely	13,894 (59.2)	6427 (66.6)^a^	1285 (70.9)^ab^
Sometimes	7118 (30.3)	2548 (26.4)^a^	429 (23.7)^a^
Often	2444 (10.4)	675 (7.0)^a^	99 (5.5)^a^

Percentages are shown in parentheses. Statistically significant and observed ±3% or more difference compared to the Absent group (^a^), and to the Prevalent group (^b^); The total percentage does not always add up to 100

### Frequency analyses of psychological factors

Participants with prevalent musculoskeletal pain and exacerbated musculoskeletal pain showed significantly greater proportions of traumatic reaction, psychological distress, and two or more of uncomfortable symptoms compared to participants without any pain (Table 3). These point-estimated odds ratios of psychological factors are shown in the Supplemental Table 2.

**Table 3:** Associated psychological factors with prevalent and exacerbated musculoskeletal pain

	Musculoskeletal pain		
	Absent	Prevalent	Exacerbated
Traumatic reaction (PCL)			
Normal	20,196 (86.1)	6643 (68.8)^a^	1000 (55.2)^ab^
44 points or over	3260 (13.9)	3007 (31.2)^a^	813 (44.8)^ab^
Psychological distress (K6)			
Normal	21,345 (91.0)	7561 (88.4)^a^	1223 (67.5)^ab^
13 points or over	2111 (9.0)	2089 (21.6)^a^	590 (32.5)^ab^
Uncomfortable symptoms			
Diarrhea/constipation	2221 (9.5)	2881 (29.9)^a^	689 (38.0)^ab^
Headache	2078 (8.9)	3060 (31.7)^a^	706 (38.9)^ab^
Dizziness	1329 (5.7)	1950 (20.2)^a^	448 (24.7)^ab^
Breathing trouble	1034 (4.4)	1792 (18.6)^a^	407 (22.4)^ab^
Palpitations	1043 (4.4)	1656 (17.2)^a^	416 (22.9)^ab^
Poor appetite	476 (2.0)	793 (8.2)^a^	234 (12.9)^ab^
Difficulty urinating	281 (1.2)	515 (5.3)^a^	140 (7.7)^a^
Number of uncomfortable symptoms			
None	17,985 (76.7)	3149 (32.6)^a^	439 (24.2)^ab^
One	2889 (12.3)	2599 (26.9)^a^	471 (26.0)^ab^
Two or more	2582 (11.0)	3902 (40.4)^a^	903 (49.8)^ab^

Percentages are shown in parentheses. Statistically significant and observed ±3% or more difference compared to the Absent group (^a^), and to the Prevalent group (^b^); The total percentage does not always add up to 100; PCL, post-traumatic stress disorder check list; K6, Kessler psychological distress scale

### Multivariable adjusted analyses of lifestyle factors

Multinomial logistic regression analysis was used to calculate multivariate adjusted odds ratios and 95% confidence intervals. After adjusting their background information, disaster experience, psychological factors, the number of uncomfortable symptoms, and each lifestyle factor (Model 2), the lifestyle factors that were significantly associated with prevalent musculoskeletal pain and exacerbated musculoskeletal pain were decreased income (prevalent: 1.13, 1.05–1.21; exacerbated: 1.29, 1.14–1.45), current heavy drinking (prevalent: 1.33, 1.21–1.47; exacerbated: 1.38, 1.14–1.68), insomnia (prevalent: 1.22, 1.15–1.29; exacerbated: 1.50, 1.36–1.65), exercising almost daily (prevalent: 0.83, 0.77–0.91; exacerbated: 0.80, 0.68–0.95), and participating in community activities often (prevalent: 0.83, 0.75–0.92; exacerbated: 0.76, 0.61–0.95) (Table 4). Shelter use (exacerbated: 1.44, 1.29–1.60) and job loss (exacerbated: 1.30, 1.16–1.47) were significantly associated only with exacerbated musculoskeletal pain.

**Table 4:** Multinomial odds ratios of associated post-disaster lifestyle factors with prevalent and exacerbated musculoskeletal pain

	Crude		Multivariable adjusted odds ratios^1^		Multivariable adjusted odds ratios^2^	
	Prevalent	Exacerbated	Prevalent	Exacerbated	Prevalent	Exacerbated
Shelter use						
Yes	**1.28**, 1.22–1.35	**2.04**, 1.85–2.25	**1.11**, 1.05–1.17	**1.57**, 1.42–1.74	1.02, 0.96–1.08	**1.44**, 1.29–1.60
Job loss						
Yes	**1.28**, 1.22–1.37	**1.92**, 1.73–2.13	**1.19**, 1.12–1/27	**1.57**, 1.40–1.76	1.03, 0.96–1.10	**1.30**, 1.16–1.47
Decreased income						
Yes	**1.19**, 1.12–1.26	**1.40**, 1.25–1.57	**1.18**, 1.11–1.26	**1.36**, 1.21–1.54	**1.13**, 1.05–1.21	**1.29**, 1.14–1.45
Smoking status						
Past	1.00, 0.94–1.06	**0.83**, 0.73–0.93	**1.15**, 1.07–1.24	1.14, 0.98–1.33	1.04, 0.96–1.12	1.02, 0.87–1.19
Current light smoking	**0.91**, 0.84–0.99	**0.73**, 0.61–0.88	**1.10**, 1.00–1.21	0.99, 0.82–1.20	1.00, 0.91–1.11	0.85, 0.70–1.04
Current heavy smoking	**0.94**, 0.86–1.02	0.86, 0.73–1.02	**1.15**, 1.04–1.27	**1.25**, 1.02–1.52	1.06, 0.95–1.18	1.11, 0.90–1.36
Drinking status						
Past	**1.19**, 1.04–1.35	1.20, 0.93–1.54	**1.21**, 1.05–1.39	**1.37**, 1.04–1.37	0.98, 0.84–1.14	1.05, 0.79–1.39
Current light drinking	**0.90**, 0.85–0.94	**0.85**, 0.77–0.94	1.05, 0.99–1.11	1.08, 0.96–1.22	**1.08**, 1.01–1.15	1.11, 0.98–1.25
Current heavy drinking	0.95, 0.88–1.03	0.94, 0.80–1.11	**1.26**, 1.15–1.38	**1.29**, 1.07–1.56	**1.33**, 1.21–1.47	**1.38**, 1.14–1.68
Insomnia						
Yes	**2.16**, 2.05–2.26	**3.22**, 2.94–3.53	**2.10**, 2.00–2.21	**2.96**, 2.70–3.25	**1.22**, 1.15–1.29	**1.50**, 1.36–1.65
Regular exercise						
1 time per week	0.95, 0.89–1.02	**0.86**, 0.74–0.99	**0.91**, 0.85–0.98	**0.83**, 0.71–0.97	0.94, 0.87–1.02	0.86, 0.73–1.00
2–4 times per week	0.98, 0.92–1.04	1.02, 0.91–1.15	**0.89**, 0.83–0.95	0.92, 0.81–1.05	**0.92**, 0.86–0.99	0.96, 0.84–1.10
Almost daily	**0.79**, 0.73–0.84	**0.71**, 0.61–0.82	**0.74**, 0.69–0.80	**0.71,** 0.60–0.83	**0.83**, 0.77–0.91	**0.80,** 0.68–0.95
Community activities						
Sometimes	**0.77**, 0.73–0.82	**0.65**, 0.58–0.73	**0.81**, 0.77–0.86	**0.72**, 0.64–0.81	**0.93**, 0.88–0.99	**0.86**, 0.76–0.98
Often	**0.60**, 0.55–0.65	**0.44**, 0.36–0.54	**0.66**, 0.60–0.72	**0.54**, 0.44–0.67	**0.83**, 0.75–0.92	**0.76**, 0.61–0.95

Odds ratios and 95% confidence intervals were shown. **Bolded numbers** mean significant association (*p* < 0.05). For each factor, the following categories were used as a reference category: ‘no’ for shelter use, job loss, decreased income, and insomnia; ‘never’ for smoking and drinking status; ‘almost not’ for regular exercise; and ‘never or rarely’ for community activities

^1^Adjustment for background information (age, sex, history of hypertension/diabetes/dyslipidemia, and educational status) and disaster experience (tsunami/indirect nuclear power plant accident, house damage, loss of close person) and each lifestyle factors; ^2^Further adjustment for psychological factors (traumatic reaction and psychological distress) and the number of uncomfortable symptoms (none, one, and two or more)

After adjustment for psychological factors and the number of uncomfortable symptoms (from Model 1 to Model 2), associations of shelter use, job loss, smoking status, past drinking, and exercising 1 or more times per week with prevalent musculoskeletal pain, that were significant in Model 1 were no longer statistically significant in Model 2. As well, associations of current heavy smoking, past drinking, exercising 1 or more times per week with exacerbated musculoskeletal pain, that were significant in Model 1 were no longer statistically significant in Model 2. Sub-analyses of pain region, sex, and age groups are shown in the Supplemental Tables 3, 4 and 5.

## Discussion

In this 1-year post-disaster population-based cross-sectional study, exercising almost daily and participating in recreational or community activities sometimes or often showed inverse associations with both prevalent and exacerbated musculoskeletal pain. In contrast, decreased income, current heavy drinking, and insomnia showed positive associations. Use of evacuation shelters or temporary housing and job loss showed positive association only with exacerbated musculoskeletal pain. These significant associations were observed even after adjustment for psychological factors and each lifestyle factor. Nevertheless, adjustment for psychological factors appeared to weaken most of those associations.

A previous study showed that the prevalence of musculoskeletal pain among evacuees at 1.5 years after the Great East Japan Earthquake disaster was 22% for low back pain and 24% for limb pain [6]. The prevalence of low back pain in our results was similar to that in the previous study, while the prevalence of limb pain seemed to be lower. This discrepancy could be due to differences in population age and the proportion of women; our population had a mean age of 61 years and the proportion of women was 52.6%, whereas the population of the previous study had a mean age of 75 years and the proportion of women was 77.5%. Musculoskeletal pain, particularly knee pain, generally increases with age and is more frequently observed in women [27]. This may explain the abovementioned differences.

The frequency of exercise and participation in recreational or community activities showed inverse associations with musculoskeletal pain; several previous studies have supported these associations. Light to moderate intensity of physical activities may reduce the risk of chronic musculoskeletal pain [7–9], with its correlation possibly associated with regular exercise and community activities in the present study. Furthermore, maintaining physical activity in post-disaster could have more increased important than before the disaster because lifestyle changes due to disaster could cause low physical activity. For example, several lifestyle factors such as shelter use and job loss (or decreased income) were likely rare before the disaster, and a 1-year post-disaster cross-sectional study of 4316 elderly people has reported that the factors of displacement from home, non-working status, and lack of social networking (Lubben’s social network scale, 11 or lower) were significantly associated with low physical activity [28]. It remains unclear if post-disaster changes in physical activity are associated with musculoskeletal pain in the present study; further research is needed.

We also need to discuss the association of insomnia with musculoskeletal pain. According to an annual longitudinal population-based study of 1746 individuals, musculoskeletal pain may increase the risk for the incidence and persistence of insomnia [11, 12], and insomnia may increase the risk for persistent musculoskeletal pain [12]. A prospective population-based cohort study using path-analysis revealed that physical limitations and reduced social participation may contribute up to 68% to the onset of insomnia in middle-aged and older adults with pain [11]. Another post-disaster cross-sectional study has shown a significant inverse association between insomnia and social networks (i.g., having friends, interaction with neighbours) [29]. To prevent the onset of insomnia in post-disaster, further research in post-disaster would be necessary; for instance, investigating potential factors associated with physical activity and social participation especially in people with musculoskeletal pain.

Association of high alcohol consumption with prevalent and exacerbated musculoskeletal pain may have been influenced by various factors. For example, one possible reason for the associations observed with alcohol consumption is that people might be injured in a fall due to impaired motor skills, which is likely to occur when blood-alcohol concentration is 0.3 mg/ml or higher [30]. Other possible reasons are chronic alcohol myopathy [31] and gout [32]. Alternatively, we should also consider that some evacuees may use alcohol for pain relief [10]. Alcohol may affect the central nervous system and temporally reduce the intensity of the pain [10].

Throughout the above results, adjustment for psychological factors weakened most of the point-estimated odds ratios (except for current heavy drinking). The changes in psychological stress may influence the prevalent and exacerbated musculoskeletal pain [13, 33], and several previous studies have shown that post-disaster lifestyle factors were associated with psychological stress [34, 35]. For example, greater psychological distress was observed among individuals who lived in temporary housing for longer periods [34]. Shelter use tends to create psychological stress due to the change in the available space, neighbours’ noise, and lack of a future housing plan [34, 35]. These results suggest that some post-disaster lifestyle factors are potentially associated with psychological stress, and such stress could be associated with prevalent and exacerbated musculoskeletal pain.

The present study had several strengths. First, to the best of our knowledge, this appears to be one of the first studies to use such a large sample size to investigate post-disaster pain epidemiology. Second, systematic recruitment and investigation were conducted after the disaster. We should also discuss the limitations of the present study. First, this cross-sectional study was conducted at one-time point (approximately 1 year after the disaster), and different social and living environment changes may have occurred with time. Thus, these findings could not be generalised to any time point after a disaster. Moreover, it is unclear whether some lifestyle factors, such as regular exercise and community activity, had continued from before the disaster. In addition, due to the nature of the cross-sectional design, the findings do not allow us to draw conclusions on the time sequence for these lifestyle factors and the musculoskeletal pain. Second, our definition of musculoskeletal pain could not assess neck pain. In above-referenced study of older population, 4–5% had neck pain [6]. Also, neck pain is one of leading cause of years lived with disability as like low back pain [3]. These prevalence and strength of association we estimated, therefore, might be underestimated. Additionally, limb pain could not discriminate among shoulder, knee, or foot pain. Pain severity (pain intensity or disability), types (nociceptive or neuropathic pain), and related pain experiences in post-disaster conditions were unclear. Third, the low response rate of the overall survey may have influenced the results. The response rate could be different depending on the exposure status of lifestyle factors; according to this, some estimated values in our results could be greater or smaller than the true values in the whole population. For example, if the response rate was higher for those who answered “yes” to participating in recreational or community activities and lower for those with musculoskeletal pain, a stronger inverse association might have been observed between them. However, we could not determine the differences between these response rates. Fourth, we did not gather information about medication use or access to hospitals. There might have been differences between participants in their medical environments. Access to pain medication and/or accessibility to a hospital after the disaster may have differed between participants and, thus, influenced our results.

## Conclusion

In this cross-sectional study after the disaster, prevalent and exacerbated musculoskeletal pain were inversely associated with exercising almost daily and participating in recreational or community activities sometimes or often, and were positively associated with decreased income, current heavy drinking, and insomnia. Besides, the use of evacuation shelters or temporary housing and job loss were positively associated only with exacerbated musculoskeletal pain. These results suggest that post-disaster lifestyle factors are potentially associated with musculoskeletal pain. To achieve better post-disaster pain management, further studies are needed to confirm the consistency of these results in other disasters and to highlight the underlying causative mechanisms.

## Supplementary information

**Additional file 1: Table S1.** Age- and sex-stratified prevalence of musculoskeletal pain after the Great East Japan Earthquake **Table S2.** Multinomial odds ratios of psychological factors with prevalent and exacerbated musculoskeletal pain **Table S3.** Multivariable adjusted multinomial odds ratios of associated post-disaster lifestyle factors with prevalent and exacerbated musculoskeletal pain stratified by pain region **Table S4.** Multivariable adjusted multinomial odds ratios of associated post-disaster lifestyle factors with prevalent and exacerbated musculoskeletal pain stratified by sex **Table S5.** Multivariable adjusted multinomial odds ratios associated post-disaster lifestyle factors with prevalent and exacerbated musculoskeletal pain stratified by age groups

## Data Availability

The datasets analysed during the present study are not publicly available because the data of the Fukushima Health Management Survey belongs to the government of Fukushima prefecture and can only be used within that organization.
