# Supplementary material for: Lifestyle factors associated with prevalent and exacerbated musculoskeletal pain after the Great East Japan Earthquake: a cross-sectional study from the Fukushima Health Management Survey
Source: BMC Public Health. 2020 May 13;20:677. doi: 10.1186/s12889-020-08764-9 (PMC7222503; doi:10.1186/s12889-020-08764-9)
Supplement: Supplementary file 1 — Additional file 1: Table S1. Age- and sex-stratified prevalence of musculoskeletal pain after the Great East Japan Earthquake Table S2. Multinomial odds ratios of psychological factors with prevalent and exacerbated musculoskeletal pain Table S3. Multivariable adjusted multinomial odds ratios of associated post-disaster lifestyle factors with prevalent and exacerbated musculoskeletal pain stratified by pain region Table S4. Multivariable adjusted multinomial odds ratios of associated post-disaster lifestyle factors with prevalent and exacerbated musculoskeletal pain stratified by sex Table S5. Multivariable adjusted multinomial odds ratios associated post-disaster lifestyle factors with prevalent and exacerbated musculoskeletal pain stratified by age groups [file 12889_2020_8764_MOESM1_ESM.docx]

**Supplemental Tables**

Supplemental Table 1: Age- and sex-stratified prevalence of musculoskeletal pain after the Great East Japan Earthquake

|  | All | Female | | | | Male | | | | |
| --- | --- | --- | --- | --- | --- | --- | --- | --- | --- | --- |
|  |  | 40-64 years | 65-74 years | 75-89 years | p-value for trend | 40-64 years | 65-74 years | 75-89 years | p-value for trend | |
| Number, persons | 34919 | 11777 | 3826 | 2553 | - | 10223 | 4045 | 2495 | - | |
| Age, years | 61, 52-70 | 53, 47-60 | 69, 67-72^*^ | 80, 77-82^*‡^ | <0.001 | 54, 49-61 | 69, 67-72^*^ | 80, 77-82^*‡^ | <0.001 | |
| Musculoskeletal pain |  |  |  |  |  |  |  |  |  | |
| Prevalent | 27.6 | 26.7 | 31.1^*^ | 36.6^*‡^ | <0.001 | 25.1 | 26.6 | 29.5^†^ | <0.001 | |
| Exacerbated | 5.2 | 5.8 | 6.7^*^ | 6.6 | 0.044 | 3.8 | 4.5 | 5.1^*^ | 0.002 | |
| Low back pain |  |  |  |  |  |  |  |  |  | |
| Prevalent | 22.2 | 21.1 | 24.7^*^ | 28.4^*†^ | <0.001 | 20.7 | 21.4 | 24.6^*†^ | <0.001 | |
| Exacerbated | 3.6 | 4.1 | 4.5 | 4.7 | 0.148 | 2.7 | 2.9 | 3.5^†^ | 0.060 | |
| Limb pain |  |  |  |  |  |  |  |  |  | |
| Prevalent | 14.6 | 13.3 | 19.1^*^ | 24.4^*†^ | <0.001 | 11.9 | 14.7^*^ | 15.5^*^ | <0.001 | |
| Exacerbated | 2.5 | 2.7 | 3.6^*^ | 3.6^*^ | 0.002 | 1.7 | 2.5^*^ | 2.8^*^ | <0.001 | |
| ^*^Significant difference compared to 40-64 years (p<0.05); ^†^Significant difference compared to 65-74 years (p<0.05); Average, lower quartile value, and upper quartile value were shown for age. | | | | | | | | | |  |

Supplemental Table 2. Multinomial odds ratios of psychological factors with prevalent and exacerbated musculoskeletal pain

|  | Crude | | Multivariable adjusted odds ratios^1^ | |
| --- | --- | --- | --- | --- |
|  | Prevalent | Exacerbated | Prevalent | Exacerbated |
| Traumatic reaction (PCL) |  |  |  |  |
| 44 points or over | **2.80**, 2.65-2.97 | **5.04**, 4.56-5.57 | **2.45**, 2.31-2.59 | **4.06**, 3.66-4.50 |
| Psychological distress (K6) |  |  |  |  |
| 13 points or over | **2.79**, 2.62-2.98 | **4.88**, 4.38-5.43 | **2.50**, 2.34-2.68 | **4.04**, 3.62-4.51 |
| Uncomfortable symptoms |  |  |  |  |
| Diarrhea/constipation | **4.07**, 3.83-4.33 | **5.86**, 5.28-6.51 | **3.83**, 3.60-4.08 | **5.23**, 4.71-5.82 |
| Headache | **4.78**, 4.49-5.08 | **6.56**, 5.91-7.29 | **4.94**, 4.63-5.27 | **6.27**, 5.62-6.99 |
| Dizziness | **4.22**, 3.91-4.54 | **5.47**, 4.85-6.16 | **3.90**, 3.62-4.21 | **4.65**, 4.11-5.26 |
| Shortness of breath  breathing trouble | **4.95**, 4.56-5.36 | **6.28**, 5.53-7.13 | **4.45**, 4.10-4.83 | **5.55**, 4.88-6.32 |
| Palpitations | **4.45**, 4.10-4.83 | **6.40**, 5.65-7.26 | **4.06**, 3.74-4.41 | **5.41**, 4.76-6.15 |
| Poor appetite | **4.32**, 3.85-4.86 | **7.15**, 6.07-8.43 | **3.90**, 3.47-4.39 | **6.08**, 5.14-7.19 |
| Pain during urination/  difficulty urinating | **4.65**, 4.01-5.39 | **6.90**, 5.60-8.50 | **4.44**, 3.82-5.16 | **6.99**, 5.63-8.66 |
| Number of uncomfortable symptoms |  |  |  |  |
| One | **5.14**, 4.81-5.48 | **6.68**, 5.83-7.65 | **5.01**, 4.69-5.35 | **6.18**, 5.39-7.09 |
| Two or more | **8.63**, 8.11-9.19 | **14.32**, 12.69-16.17 | **8.25**, 7.74-8.79 | **12.53**, 11.08-14.18 |
| **Bolded numbers** mean significant association (p < 0.05). For each factor, the following categories were used as a reference category: ‘normal’ for traumatic reaction and psychological distress; ‘none’ for uncomfortable symptoms.  ^1^Adjustment for background information (age, sex, history of hypertension/diabetes/dyslipidemia, and educational status) and disaster experience (tsunami/indirect nuclear power plant accident, house damage, loss of close person) and each lifestyle factors. | | | | |

Supplemental Table 3: Multivariable adjusted multinomial odds ratios of associated post-disaster lifestyle factors with prevalent and exacerbated musculoskeletal pain stratified by pain region

|  | Low back pain | | Limb pain | |
| --- | --- | --- | --- | --- |
|  | Prevalent | Exacerbated | Prevalent | Exacerbated |
| Shelter use |  |  |  |  |
| Yes | 1.01, 0.95-1.08 | **1.36**, 1.20-1.54 | 1.00, 0.94-1.07 | **1.50**, 1.30-1.73 |
| Job loss |  |  |  |  |
| Yes | 1.02,0.95-1.09 | **1.26**, 1.10-1.44 | **1.13**, 1.04-1.22 | **1.35**, 1.16-1.59 |
| Decreased income |  |  |  |  |
| Yes | **1.13**, 1.05-1.21 | **1.30**, 1.13-1.50 | 1.07, 0.99-1.16 | **1.28**, 1.08-1.51 |
| Smoking status |  |  |  |  |
| Past | 1.07, 0.99-1.17 | 1.04, 0.87-1.24 | 1.01, 0.92-1.12 | 0.99, 0.80-1.22 |
| Current light smoking | 1.04, 0.94-1.16 | 0.89, 0.71-1.12 | 1.03, 0.91-1.16 | 0.82, 0.62-1.09 |
| Current heavy smoking | 1.09, 0.98-1.22 | 1.22, 0.97-1.54 | 1.00, 0.88-1.14 | 0.87, 0.64-1.17 |
| Drinking status |  |  |  |  |
| Past | 0.98, 0.84-1.15 | 1.11, 0.80-1.54 | 0.96, 0.81-1.15 | 1.03, 0.71-1.49 |
| Current light drinking | 1.05, 0.99-1.13 | 1.14, 0.99-1.31 | **1.09**, 1.01-1.18 | 1.07, 0.90-1.26 |
| Current heavy drinking | **1.34**, 1.21-1.49 | **1.43**, 1.15-1.79 | **1.21**, 1.07-1.36 | 1.20, 0.92-1.56 |
| Insomnia |  |  |  |  |
| Yes | **1.21**, 1.14-1.28 | **1.53**, 1.37-1.71 | **1.22**, 1.14-1.31 | **1.36**, 1.20-1.56 |
| Regular exercise |  |  |  |  |
| 1 time per week | 0.94, 0.86-1.03 | 0.85, 0.71-1.03 | 0.92, 0.84-1.02 | 0.87, 0.70-1.08 |
| 2-4 times per week | **0.92**, 0.95-0.99 | 0.94, 0.80-1.10 | **0.91**, 0.83-0.99 | 1.08, 0.90-1.29 |
| Almost daily | **0.85**, 0.78-0.93 | 0.89, 0.73-1.07 | **0.87**, 0.79-0.96 | **0.76**, 0.61-0.96 |
| Community activities |  |  |  |  |
| Sometimes | 0.96, 0.90-1.02 | 0.87, 0.76-1.01 | 0.94, 0.87-1.02 | 0.86, 0.73-1.02 |
| Often | **0.86**, 0.77-0.96 | 0.77, 0.59-1.00 | **0.88**, 0.77-0.99 | 0.90, 0.67-1.21 |
| **Bolded numbers** mean significant association (p < 0.05).For each factor, the following categories were used as a reference category: ‘no’ for shelter use, having job loss, decreased income, and insomnia; ‘never’ for smoking and drinking status; ‘almost not’ for regular exercise; and ‘never or rarely’ for community activities. All estimated odds ratios were adjustment for background information (age, sex, history of hypertension/diabetes/dyslipidemia, and educational status), disaster experience (tsunami/indirect nuclear power plant accident, house damage, loss of close person), psychological factors (traumatic reaction and psychological distress) and the number of uncomfortable symptoms (none, one, and two or more), and each lifestyle factors. | | | | |

Supplemental Table 4: Multivariable adjusted multinomial odds ratios of associated post-disaster lifestyle factors with prevalent and exacerbated musculoskeletal pain stratified by sex

|  | Female | | Male | |
| --- | --- | --- | --- | --- |
|  | Prevalent | Exacerbated | Prevalent | Exacerbated |
| Shelter use |  |  |  |  |
| Yes | 1.01, 0.93-1.08 | **1.46**, 1.27-1.67 | 1.05, 0.96-1.14 | **1.40**, 1.18-1.66 |
| Job loss |  |  |  |  |
| Yes | 1.03,0.94-1.13 | **1.27**, 1.09-1.48 | 1.06, 0.96-1.18 | **1.41**, 1.17-1.70 |
| Decreased income |  |  |  |  |
| Yes | **1.11**, 1.00-1.22 | **1.26**, 1.07-1.49 | **1.12**, 1.02-1.23 | **1.28**, 1.07-1.54 |
| Smoking status |  |  |  |  |
| Past | 1.07, 0.92-1.24 | 1.05, 0.81-1.37 | 1.05, 0.95-1.16 | 1.00, 0.82-1.24 |
| Current light smoking | 1.05, 0.90-1.22 | 0.99, 0.75-1.30 | 0.98, 0.85-1.12 | 0.75, 0.55-1.01 |
| Current heavy smoking | 1.09, 0.85-1.38 | 1.21, 0.81-1.79 | 1.01, 0.89-1.14 | 1.02, 0.79-1.32 |
| Drinking status |  |  |  |  |
| Past | 1.17, 0.86-1.60 | 0.97, 0.56-1.69 | 0.99, 0.83-1.20 | 1.16, 0.82-1.65 |
| Current light drinking | 1.04, 0.96-1.14 | 1.07, 0.91-1.25 | 1.17, 1.05-1.29 | 1.22, 0.99-1.51 |
| Current heavy drinking | **1.33**, 1.05-1.69 | 1.34, 0.89 -2.00 | **1.37**, 1.21-1.54 | **1.48**, 1.16-1.90 |
| Insomnia |  |  |  |  |
| Yes | **1.21**, 1.12-1.30 | **1.48**, 1.31-1.68 | **1.24**, 1.13-1.35 | **1.53**, 1.30-1.80 |
| Regular exercise |  |  |  |  |
| 1 time per week | 0.92, 0.82-1.03 | **0.80**, 0.65-0.99 | 0.97, 0.86-1.09 | 0.95, 0.74-1.21 |
| 2-4 times per week | **0.90**, 0.82-0.99 | 0.97, 0.82-1.16 | 0.96, 0.86-1.07 | 0.92, 0.74-1.15 |
| Almost daily | **0.83**, 0.74-0.93 | 0.86, 0.69-1.07 | **0.86**, 0.76-0.96 | **0.73**, 0.57-0.94 |
| Community activities |  |  |  |  |
| Sometimes | 0.93, 0.86-1.02 | 0.86, 0.74-1.01 | 0.94, 0.86-1.03 | 0.86, 0.71-1.05 |
| Often | **0.81**, 0.70-0.94 | **0.70**, 0.51-0.95 | **0.85**, 0.74-0.98 | 0.84, 0.61-1.16 |
| **Bolded numbers** mean significant association (p < 0.05). For each factor, the following categories were used as a reference category: ‘no’ for shelter user, job loss, decreased income, and insomnia; ‘never’ for smoking and drinking status; ‘almost not’ for regular exercise; and ‘never or rarely’ for community activities. All estimated odds ratios were adjustment for background information (age, history of hypertension/diabetes/dyslipidemia, and educational status), disaster experience (tsunami/indirect nuclear power plant accident, house damage, loss of close person), psychological factors (traumatic reaction and psychological distress) and the number of uncomfortable symptoms (none, one, and two or more), and each lifestyle factors. | | | | |

Supplemental Table 5: Multivariable adjusted multinomial odds ratios associated post-disaster lifestyle factors with prevalent and exacerbated musculoskeletal pain stratified by age groups

|  | 40-64 years | | 65-89 years | |
| --- | --- | --- | --- | --- |
|  | Prevalent | Exacerbated | Prevalent | Exacerbated |
| Shelter use |  |  |  |  |
| Yes | 1.05, 0.98-1.13 | **1.49**, 1.30-1.71 | 0.97, 0.89-1.07 | **1.34**, 1.13-1.58 |
| Job loss |  |  |  |  |
| Yes | 1.04,0.96-1.13 | **1.35**, 1.17-1.55 | 1.04, 0.91-1.19 | **1.25**, 1.00-1.56 |
| Decreased income |  |  |  |  |
| Yes | **1.13**, 1.04-1.22 | **1.35**, 1.16-1.56 | 1.09, 0.96-1.24 | 1.15, 0.92-1.44 |
| Smoking status |  |  |  |  |
| Past | 1.09, 0.99-1.21 | 1.01, 0.83-1.24 | 0.99, 0.87-1.13 | 1.04, 0.81-1.34 |
| Current light smoking | 1.03, 0.92-1.16 | 0.81, 0.64-1.03 | 0.96, 0.79-1.17 | 0.95, 0.65-1.40 |
| Current heavy smoking | 1.07, 0.94-1.20 | 1.08, 0.85-1.37 | 0.95, 0.75-1.20 | 1.08, 0.69-1.70 |
| Drinking status |  |  |  |  |
| Past | 0.89, 0.70-1.13 | 1.26, 0.84-1.88 | 1.15, 0.94-1.41 | 0.94, 0.63-1.40 |
| Current light drinking | 1.06, 0.98-1.15 | 1.09, 0.94-1.27 | **1.16**, 1.04-1.30 | 1.16, 0.95-1.43 |
| Current heavy drinking | **1.26**, 1.12-1.41 | **1.45**, 1.16-1.81 | **1.46**, 1.20-1.77 | 1.17, 0.79-1.71 |
| Insomnia |  |  |  |  |
| Yes | **1.25**, 1.17-1.34 | **1.46**, 1.29-1.66 | **1.16**, 1.06-1.28 | **1.55**, 1.32-1.83 |
| Regular exercise |  |  |  |  |
| 1 time per week | 0.93, 0.84-1.03 | 0.82, 0.66-1.00 | 0.93, 0.81-1.07 | 0.94, 0.72-1.21 |
| 2-4 times per week | 0.94, 0.85-1.03 | 0.88, 0.73-1.07 | **0.89**, 0.79-0.99 | 1.06, 0.86-1.31 |
| Almost daily | 0.91, 0.81-1.03 | 0.81, 0.63-1.04 | **0.78**, 0.69-0.88 | 0.84, 0.66-1.06 |
| Community activities |  |  |  |  |
| Sometimes | **0.89**, 0.82-0.97 | **0.81**, 0.69-0.96 | 0.99, 0.90-1.09 | 0.93, 0.78-1.12 |
| Often | **0.84**, 0.72-0.97 | 0.70, 0.49-1.01 | **0.84**, 0.73-0.97 | 0.81, 0.61-1.08 |
| For each factor, the following categories were used as a reference category: ‘no’ for shelter use, job loss, decreased income, and insomnia; ‘never’ for smoking and drinking status; ‘almost not’ for regular exercise; and ‘never or rarely’ for community activities. All estimated odds ratios were adjustment for background information (age, sex, history of hypertension/diabetes/dyslipidemia, and educational status), disaster experience (tsunami/indirect nuclear power plant accident, house damage, loss of close person), psychological factors (traumatic reaction and psychological distress) and the number of uncomfortable symptoms (none, one, and two or more), and each lifestyle factors. | | | | |
